# ATP Supply from Cytosol to Mitochondria Is an Additional Role of Aerobic Glycolysis to Prevent Programmed Cell Death by Maintenance of Mitochondrial Membrane Potential

**DOI:** 10.3390/metabo15070461

**Published:** 2025-07-07

**Authors:** Akane Sawai, Takeo Taniguchi, Kohsuke Noguchi, Taisuke Seike, Nobuyuki Okahashi, Masak Takaine, Fumio Matsuda

**Affiliations:** 1 Department of Bioinformatic Engineering, Graduate School of Information Science and Technology, The University of Osaka, 1-5 Yamadaoka, Suita 565-0871, Japantakeo_taniguchi@ist.osaka-u.ac.jp (T.T.);; 2 Shimadzu Analytical Innovation Research Laboratory, Graduate School of Engineering, The University of Osaka, 2-1 Yamadaoka, Suita 565-0871, Japan; 3 Gunma University Initiative for Advanced Research (GIAR), Gunma University, 4-2 Aramaki-machi, Maebashi 371-0044, Japan; masaktakaine@gunma-u.ac.jp; 4 Institute for Molecular and Cellular Regulation (IMCR), Gunma University, 4-2 Aramaki-machi, Maebashi 371-0044, Japan

**Keywords:** aerobic glycolysis, breast cancer cells, *Saccharomyces cerevisiae*, metabolic rewiring, mitochondrial membrane potential, reverse mode of H^+^/ATPase, programmed cell death

## Abstract

Eukaryotic cells generate ATP primarily via oxidative and substrate-level phosphorylation. Despite the superior efficiency of oxidative phosphorylation, eukaryotic cells often use both pathways as aerobic glycolysis, even in the presence of oxygen. However, its role in cell survival remains poorly understood. **Objectives:** In this study, aerobic glycolysis was compared between the Warburg effect in breast cancer cells (MCF7) and the Crabtree effect in a laboratory strain of *Saccharomyces cerevisiae* (S288C). **Methods:** The metabolic adaptations of MCF7 and S288C cells were compared following treatment with electron transport chain inhibitors, including FCCP, antimycin A, and oligomycin. **Results:** MCF7 and S288C cells exhibited strikingly similar metabolic rewiring toward substrate-level phosphorylation upon inhibitor treatment, suggesting that mitochondrial oxidative phosphorylation and cytosolic substrate-level phosphorylation communicate through a common mechanism. Measurement of mitochondrial membrane potential (MMP) and ATP concentrations further indicated that cytosolic ATP was transported into the mitochondria under conditions of reduced electron transport chain activity. This ATP was likely utilized in the reverse mode of H^+^/ATPase to maintain MMP, which contributed to the avoidance of programmed cell death. **Conclusions**: These results suggest that the ATP supply to mitochondria plays a conserved role in aerobic glycolysis in yeast and mammalian cancer cells. This mechanism likely contributes to cell survival under conditions of fluctuating oxygen availability.

## 1. Introduction

Eukaryotic cells primarily produce ATP via two pathways: oxidative phosphorylation in the mitochondria and substrate-level phosphorylation in the cytosol [1]. Oxidative phosphorylation efficiently generates 32–36 ATP molecules through the complete oxidation of one glucose molecule. Most ATP are regenerated by H^+^/ATPase in the electron transport chain by consuming mitochondrial membrane potential (MMP). In contrast, substrate-level phosphorylation produces only two ATP molecules during the conversion of glucose to lactate or ethanol without requiring oxygen.

Despite the high efficiency of oxidative phosphorylation, eukaryotic cells often utilize both pathways simultaneously, even in the presence of oxygen. This metabolic state is known as aerobic glycolysis. For instance, cancer cells preferentially rely on substrate-level phosphorylation, a phenomenon known as the Warburg effect, which is considered a hallmark of cancer [2,3]. However, oxidative phosphorylation remains active in many cultured cancer cells, with some cells supplying more than 50% of their ATP via oxidative phosphorylation under aerobic conditions [4,5,6].

The Warburg effect in human cancer cells is often compared to the Crabtree effect in *Saccharomyces cerevisiae*, a eukaryotic model organism [1,7]. Crabtree-positive *S. cerevisiae* has evolved the ability to perform substrate-level phosphorylation even under aerobic conditions [8]. From a metabolic perspective, cancer cells and *S. cerevisiae* share aerobic glycolysis as a common strategy for ATP regeneration. Thus, a comparison of these organisms is expected to provide clues to understanding the role of the Warburg effect in cancer development and the evolutionary significance of the Crabtree effect in *S. cerevisiae*.

One question regarding aerobic glycolysis is why cancer cells and *S. cerevisiae* deliberately rely on inefficient substrate-level phosphorylation, even under aerobic conditions. Several rational roles for aerobic glycolysis have been proposed, including rapid ATP and substrate supply to support rapid cell proliferation in cancer cells [9], modulation of the cancer microenvironment through lactate excretion [10], and a possible advantage related to the prevalence from an upper limit on Gibbs energy dissipation [11]. These findings suggest that aerobic glycolysis played a multifaceted role in the evolutionally context of eukaryotic cells. Furthermore, the widespread occurrence of aerobic glycolysis in diverse cells and species implies a conserved and fundamental role for aerobic glycolysis shared by cancer and yeast cells.

The basic role of aerobic glycolysis may result from an interplay between oxidative phosphorylation in the mitochondria and substrate-level phosphorylation in the cytosol. However, whether metabolic regulatory mechanisms are conserved between mammalian cancer cells and unicellular *S. cerevisiae* remains unclear. Previous studies have demonstrated that complex regulatory mechanisms govern the interactions between oxidative and substrate-level phosphorylation, as perturbations in one pathway can lead to compensatory changes in another [12,13]. For example, in melanoma cells, a low-dose treatment with complex I inhibitors activates substrate-level phosphorylation [14]. Similarly, in *S. cerevisiae*, increased NADH oxidation reduces the overflow metabolism [15]. Additionally, the overexpression of Hap4, a positive transcriptional regulator of respiratory metabolism genes, modulated the flux distribution between respiration and fermentation in *S. cerevisiae* [16]. If the regulatory mechanisms driving aerobic glycolysis are shared between human cancer cells and *S. cerevisiae*, this suggests that the role of aerobic glycolysis may be more intrinsic to metabolic functions and essential for the survival of eukaryotic cells.

This study investigated the physiological significance of aerobic glycolysis and the interaction mechanisms between the cytoplasm and mitochondria during aerobic glycolysis. We employed two complementary approaches to achieve these research objectives. First, we conducted a comparative analysis of aerobic glycolysis in human breast cancer cells (MCF7) and an *S. cerevisiae* laboratory strain (S288C). MCF7 cells were selected because of their characteristic ATP production profile, with approximately 30% of the ATP generated through oxidative phosphorylation under aerobic conditions [17]. Second, we examined the cytoplasm–mitochondrial interactions during small perturbations induced by low doses of three electron transport chain inhibitors: FCCP, antimycin A, and oligomycin. These results demonstrate remarkably conserved metabolic responses to oxidative phosphorylation dysfunction in cancer cells and yeast at the flux, metabolome, and gene expression levels. Furthermore, our data indicates that upon suppression of electron transport chain activity, cytosolic ATP is transported to the mitochondria and utilized in the reverse mode of H^+^/ATPase to maintain MMP. Given that decreased MMP levels promote programmed cell death, this mechanism likely plays a crucial role in cell survival against programmed cell death under variable oxygen availability.

## 2. Materials and Methods

*S. cerevisiae* strain and culture conditions: Two laboratory strains of *S. cerevisiae* S288C (BY27002) and BY18615 were purchased from the National BioResource Project (NBRP, Shizuoka, Japan). The *S. cerevisiae* strains and plasmids used in this study are listed in Table S1. *S. cerevisiae* cells were inoculated into 5 mL of yeast extract peptone dextrose (YPD) medium (1% Bacto yeast extract, 2% Bacto peptone, 2% glucose) in a test tube and cultured with shaking at 30 °C and 150 rpm for 24 h using a Personal shaker-11 (TAITEC Co., Saitama, Japan). For pre-culture, cells were transferred into 50 mL of synthetic dextrose (SD) medium (0.5% glucose, 0.67% yeast nitrogen base without amino acids) in a 200 mL baffled flask and cultured overnight with shaking at 30 °C and 120 rpm using a Bio Shaker (GBR-200, TAITEC). For the main culture, cells were inoculated into 5 mL of SD medium in a CELLSTAR 6-well plate with low evaporation lid (Greiner Bio-one, Kremsmünster, Austria) and cultured with shaking at 30 °C and 185 rpm using a Synergy HTX multi-mode plate reader (BioTek, Winooski, VT, USA.). Cell concentration was determined by measuring the optical density at 600 nm (OD_600_) using a UV-visible spectrophotometer (UV-1280, Shimadzu, Kyoto, Japan). The initial OD_600_ values of the pre- and main cultures were 0.05.

Cancer cell and culture conditions: The MCF7 human breast carcinoma cell line was purchased from the Riken Bioresource Center (BRC). Cells (3.6 × 10^5^ cells) were seeded in 5 mL of medium in 60 mm (diameter) plates and cultured at 37 °C with 5% CO_2_. For passage cultures, Dulbecco’s modified Eagle’s medium (DMEM) containing 10% fetal bovine serum (FBS) and 1% penicillin/streptomycin (Fujifilm Wako Pure Chemical Co., Osaka, Japan) was used. For metabolic analysis, cells were cultured in 5 mL DMEM without glucose, L-glutamine, phenol red, sodium pyruvate, or sodium bicarbonate (Sigma Aldrich, St. Louis, MO, USA) and supplemented with 20 mM glucose, 2 mM glutamine, 3.7 g/L sodium bicarbonate, and 10% dialyzed FBS (Lot No. 2133990, Gibco, Thermo Fisher Scientific, Waltham, MA, USA). A glucose concentration of 10 mM was employed for medium component analysis, and cell numbers were measured 10 times at any location within the dish using CKX-CCSW (OLYMPUS, Tokyo, Japan), with a culture area of 2100 mm^2^, objective lens magnification of 10×, and adapter magnification of 0.5×.

Measurement of extracellular metabolite concentration: For extracellular metabolite measurements, culture medium (0.1–1.0 mL) was collected, mixed with an equal volume of 20 mM pimelate solution (internal standard), and filtered through a filter cartridge (0.45 µm pore size). Extracellular metabolite concentrations were determined using high-performance liquid chromatography (HPLC; Prominence, Shimadzu) equipped with a refractive index detector (RI) and an Aminex HPX-87H column (Bio-Rad, Hercules, CA, USA), as previously described [18].

Inhibitor treatment: This study utilized three inhibitors—FCCP [Carbonyl cyanide 4-(trifluoromethoxy) phenylhydrazone] (Sigma-Aldrich), antimycin A (from *Streptomyces* sp.; Sigma-Aldrich), and oligomycin (Fujifilm Wako). For MCF7 treatments, stock solutions were prepared as 5 mM FCCP in DMSO, 100 µM antimycin A in ethanol, and 100 µM oligomycin in ethanol. For *S. cerevisiae*, the stock solutions were 5 mM FCCP in DMSO, 40 mM antimycin A in DMSO, and 40 mM oligomycin in DMSO. All stock solutions were added to the medium at a final concentration of 0.1% (*v*/*v*).

Metabolome analysis of intracellular metabolites: *S. cerevisiae* cells (approximately OD_600_ × mL = 10) were collected from the main culture by the filtration method (PTFE membrane filter: 0.45 µm pore size, 47 mm diameter; Omnipore, Merck Millipore, Kenilworth, NJ, USA). The cells were immersed in 1.6 mL of methanol containing an internal standard (20 µM D-camphor sulfonate) and stored at −80 °C. Intracellular metabolites were extracted using the methanol/chloroform/water method with addition of 640 µL of Milli-Q water and 1.6 mL of chloroform. For MCF7 cells, the medium was quickly removed from the dish, and the cells were washed with 1 mL of phosphate-buffered saline (PBS). Immediately after washing, 800 μL of −80 °C cooled methanol was spread over the entire dish to stop metabolism. The cells were scraped on ice using a cell scraper, collected with the methanol into tubes, and stored at −80 °C. Intracellular metabolites were similarly extracted using the methanol/chloroform/water method, with 320 µL of Milli-Q water and 0.8 mL of chloroform. The resulting mixture was centrifuged (2580× *g*, 4 °C, 20 min), and 250 µL of the upper phase was aliquoted into six or three Eppendorf tubes, dried under vacuum using a Speed Vac (Thermo Fisher Scientific). Metabolome data were obtained via ion-pair liquid chromatography–triple quadrupole mass spectrometry (LC-MS/MS) and gas chromatography–quadrupole mass spectrometry (GC-MS) were performed, as in the previously reported method [19]. For GC-MS analysis, dried samples were derivatized by adding 100 μL each of methoxyamine hydrochloride in pyridine (40 mg/mL) and *N*-methyl-*N*-(*tert*-butyldimethylsilyl)trifluoroacetamide (MTBSTFA) with 1% *tert*-butyldimetheylchlorosilane (TBDMCS) (Thermo Fisher). All data were processed using the LabSolution software (version 5.1, Shimadzu).

Quantitative PCR analysis: *S. cerevisiae* cells (approximately OD_600_ × mL = 2) were collected from the main culture by centrifugation at 5000× *g* for 10 min. Following removal of the supernatant, the cell wall was disrupted by adding 10 μL of 1000 U/mL zymolyase (Nacalai Tesque, Kyoto, Japan) solution, 40 μL of 500 mM EDTA, and 60 μL of sterile water, followed by incubation at 37 °C for 30 min. For MCF7 cells, the medium was removed, and cells were collected following the manufacture’s protocol. RNA was extracted from the collected cells using the Nucleo SpinR RNA kit (Takara Bio, Shiga, Japan). cDNA was synthesized using the PrimeScript RT Master Mix (Perfect Real Time) kit (Takara Bio) using the TaKaRa PCR Thermal Cycler Dice (Takara Bio) (37 °C for 15 min, 85 °C for 5 s). qPCR was conducted on a StepOnePlus Real-Time PCR System (Applied Biosystems, Waltham, MA, USA) using TB GreenR Premix Ex TaqII (Tli RNaseH Plus) (Takara Bio). The amplification conditions were Stage 1: 95 °C for 5 s and Stage 2: 95 °C for 5 s and 60 °C for 30 s, repeated for 40 cycles. The primers used are listed in Table S2. Relative expression levels were calculated using the ΔΔCt method [20], using *ACT1* and *GAPDH* as internal controls for *S. cerevisiae* and MCF7 cells, respectively.

Measurement of mitochondrial membrane potential: *S. cerevisiae* culture broth (approximately OD_600_ × mL = 1) was collected and treated with MitoTracker Red CM-H2Xros (Invitrogen, Carlsbad, CA, USA) at a final concentration of 400 nM. Following additional cultivation with shaking at 30 °C for 30 min, the medium was removed, and the cells were resuspended in 30 μL of PBS. The suspended cells were then placed on glass plates. For the analysis of MCF7 cells, 1.5 × 10^5^ cells were seeded in 1.5 mL of passage medium in a 35 mm glass-bottom dish (Matsunami Glass Ind., Osaka, Japan). After overnight cultivation, the medium was replaced with metabolic analysis medium containing 0.1% (*v*/*v*) inhibitors. Following 3 h of culture, 750 μL of supernatant was removed, and 750 μL of MitoTracker Red CM-H2Xros (400 nM, diluted in the metabolic analysis medium). After 30 min of incubation, the medium was removed and replaced with 1 mL of PBS. Fluorescence microscopy observation was performed using an IX71 Fluorescence Microscope (Olympus) equipped with a U-MWIG3 filter (Ex530-550/DM570/BA575-625, Olympus) and an ORCA-spark digital CMOS camera C11578-36U (Hamamatsu Photonics, Aichi, Japan). All images were processed and analyzed using CellSens (Olympus) and Fiji [21].

Measurement of mitochondrial membrane potential under hypoxic conditions: For *S. cerevisiae*, cells in pre-culture were suspended in 1 mL SD medium with OD_600_ = 0.05 in 1.5 mL microcentrifuge tubes (Eppendorf, Hamburg, Germany) and incubated at 30 °C for 24 h in an AnaeroPack rectangular jar standard type (Sugiyama-Gen Co., Tokyo, Japan) containing an AnaeroPack-Kenki oxygen scavenger (Sugiyama-Gen). Following treatment with MitoTracker Red CM-H2Xros at a final concentration of 400 nM, the cell suspension was transferred to a 35 mm glass-bottom dish (AGC Techno glass, Co., Shizuoka, Japan) and cultured for 30 min in a low-oxygen incubator CH-070A (BLAST, Kanagawa, Japan). Fluorescence observations were made with the dish remaining in the culture chamber. For MCF7 cells (1.5 × 10^5^ cells) were seeded in 1.5 mL of passage medium in a 35 mm glass-bottom dish and cultured overnight to allow attachment of the cells to the bottom of the dish. The medium was replaced with the metabolic analysis medium containing 0.1% (*v*/*v*) inhibitors and cultured at 5% CO_2_, 1% O_2_, and 37 °C for 24 h using a low-oxygen incubator CH-070A. An AnaeroPack-Kenki was used as an oxygen scavenger. After treatment with MitoTracker Red CM-H2Xros, as described above, fluorescence observations were conducted while maintaining the hypoxic state by keeping the dish in the low-oxygen incubator.

Construction of *S. cerevisiae* strains expressing cytoplasmic and mitochondria-localized QUEEN-2m: Based on the amino acid sequence of the intracellular ATP sensor QUEEN-2m [22], a codon-optimized ORF was synthesized and cloned into the pGEM-T Easy vector (Promega, Madison, WI, USA). The pGEM-T Easy-QUEEN-2m plasmid and a multicopy yeast expression plasmid pGK426 [23] were digested with SalI and EcoRI and the resulting fragments were ligated to construct pGK426-QUEEN-2m. To create the mitochondria-localized QUEEN-2m expression plasmid (pAS2, Figure S1), inverse PCR was performed using the pGK426-QUEEN-2m as the template. Primers (Queen-2m_Cox4N_invF and Queen-2m_Cox4N_invR; Table S3) were designed to amplify outward from the N-terminus of the QUEEN-2m gene, including the sequence encoding the mitochondrial targeting signal derived from *S. cerevisiae* Cox4. The obtained linear DNA fragment was phosphorylated using T4 Polynucleotide Kinase (New England Labs, Ipswich, MA, USA) and ligated using T4 DNA Ligase and T4 Polynucleotide Kinase (New England Labs) with T4 DNA Ligase (New England Labs). The circularized plasmids were transformed into *Escherichia. coli* DH5α and plated on LB agar containing ampicillin (100 μg/mL). Plasmid DNA was extracted from colonies grown on the selection plates, and the presence of the correctly introduced Cox4 pre-sequence was confirmed by sequencing. The construct was designed to create a circularized plasmid pAS1. Furthermore, to construct pAS2, which harbors two ORFs of QUEEN-2m, inverse PCR was performed on pAS1 using primers (AS1 fusion-F1 and AS1 fusion-R1; Table S3), starting 670 bp upstream of the PGK1 terminator. An additional ORF was obtained by PCR using pAS1 as a template and primers AS1 QUEEN-F1 and AS1 QUEEN-R1 (Table S3). The obtained vector and fragment were fused using the Gibson Assembly method with the Gibson Assembly Master Mix (New England Biolabs, Ipswich, MA, USA) and transformed into *E. coli* DH5α. Plasmid DNA was extracted from colonies grown on LB agar containing ampicillin (100 µg/mL), and sequencing confirmed the presence of two copies of the QUEEN-2m ORFs in the construct. The *S. cerevisiae* strain BY18615 (MATα, ura3) (purchased from the National BioResource Project, NBRP) was transformed with the pGK426-QUEEN-2m and pAS2 plasmids using the standard. The lithium acetate method was used as the transformation method.

Measurement of ATP concentration in cytoplasm and mitochondria using QUEEN-2m expressed *S. cerevisiae* strains: The analysis was performed following a previously described protocol [24]. *S. cerevisiae* cells were anchored onto glass plates coated with concanavalin A. A 35 mm glass-bottom dish was prepared by soaking with 60 μL of concanavalin A solution (2 mg/mL, Sigma) for 5 min. The concanavalin A solution was then removed, and the dish was washed twice with 100 μL of sterile water. From a log-phase pre-culture of *S. cerevisiae* strain expressing QUEEN-2m, 100 μL of culture broth was collected and placed on the prepared dish for 5 min. After carefully removing 80 μL of the medium to avoid detaching the cells, 80 μL of fresh SD medium was added, gently suspended five times, and removed. This process was repeated twice, followed by addition of 80 μL of fresh SD medium. Fluorescence observations were made using an inverted fluorescence microscope (ECLIPSE TE2000-E, Nikon, Tokyo, Japan) equipped with a 60× oil immersion objective lens (Plan Apo 60 × 1.40 Oil Ph3 DM), a GFP filter set (Ex457-481/DM495/BA502-538, Nikon), a QUEEN filter set (Ex393-425/DM506/BA516-556, Semrock), and an EM-CCD camera IXON (Nikon). During fluorescence imaging, the sample was maintained at 30 °C using a microscope temperature control system (TOKAI HIT, Shizuoka, Japan). To minimize sample fading, the exposure time was set to 50 ms for both GFP and QUEEN fluorescence. Images were captured every 10 min, and the response after inhibitor treatment was observed continuously for 120 min. The fluorescence image data were analyzed using Fiji [21]. All images were converted to 32 bit format after background subtraction using the rolling-ball algorithm. A threshold level for detecting cell regions was set using the IsoData algorithm, with pixel values outside the detected cell regions assigned as NaN values. The QUEEN/GFP ratio was determined using processed fluorescence images of GFP and QUEEN. For each captured image, cell regions were extracted, and the mean intensity ratio was calculated for each cell type. For time-course data, stacks were created for both GFP and QUEEN fluorescence images, and image processing was performed for each stack. The LPixel plugin was used to correct any image shifts during continuous shooting.

## 3. Results

### 3.1. Similar Metabolic Rewiring upon Respiratory Chain Inhibitor Treatment Between Breast Cancer Cells (MCF7) and S. Cerevisiae (S288C)

**Figure 1:**
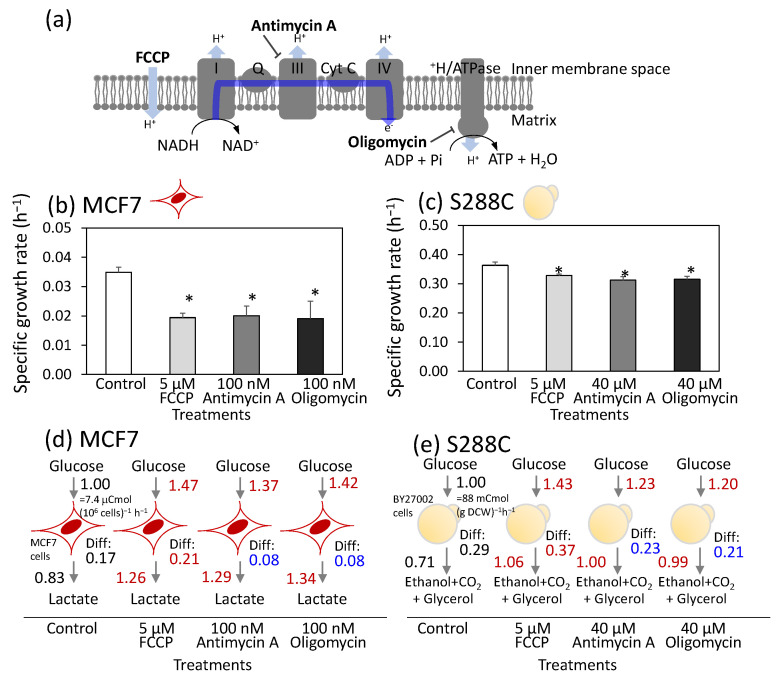
Similar metabolic rewiring upon respiratory chain inhibitor treatment between human breast cancer cells, MCF7, and *Saccharomyces cerevisiae,* S288C. (**a**) Targets of respiratory chain inhibitors in human mitochondria. (**b**,**c**) Effect of FCCP, antimycin A, and oligomycin treatment on specific growth rates of (**b**) MCF7 and (**c**) S288C. All results were obtained from triplicate cultures and described as mean ± SD. Asterisks indicate *p*-value < 0.05 by two-sided *t*-test. (**d**,**e**) Metabolic rewiring of (**d**) MCF7 and (**e**) S288C upon the inhibitor treatment. Carbon balances were calculated from the specific rates for glucose consumption and product excretion. All values represent the carbon flux level relative to the carbon uptake level of the control culture. Differences in carbon balance (Diff) between glucose uptake and product excretion are also shown. All results were obtained from triplicate cultures and described as mean values. Red and blue letters indicate a significant increase and decrease, respectively, with *p*-value < 0.05 by two-sided *t*-test compared to the control. Red (MCF7) and yellow (S288C) illustrations indicate the cell types used in each experiment.

To evaluate the effect of the respiratory chain inhibitor on cell proliferation, MCF7 human breast cancer cells were seeded in a dish containing passage medium and cultured for 15 h. Once the cells adhered to the dish, the medium was replaced with metabolic analysis medium supplemented with specific respiratory chain inhibitors. In this study, FCCP, antimycin A, and oligomycin were used to disrupt oxidative phosphorylation-dependent ATP synthesis (Figure 1a). FCCP functions as an uncoupler by transporting protons across the mitochondrial inner membrane and dissipating the proton gradient. Antimycin A inhibits the electron transport from cytochrome b to cytochrome complex III. Oligomycin inhibits mitochondrial H^+^/ATPases and blocks ATP synthesis and hydrolysis.

The specific growth rate of the control MCF7 culture during the log phase was 0.035 ± 0.002 h^−1^ (Figure 1b). The specific growth rate decreased to approximately half that of the control culture when treated with 5 μM FCCP, 100 nM antimycin A, and 100 nM oligomycin (Figure 1b). The concentrations of FCCP, antimycin A, and oligomycin used in this study were similar to those in the standard protocol for the FluxAnalyzer (FCCP: 0.125–2.0 µM, antimycin A: 500 nM, and oligomycin: 1500 nM) [25]. These results suggest that the respiratory chain was partially but not completely inhibited by these inhibitor concentrations.

For comparison, cells of a laboratory strain of *S. cerevisiae* (S288C) were batch-cultured under aerobic conditions in 6-well plates. The specific growth rate of the control culture was determined to be 0.37 ± 0.01 h^−1^ from the growth curve (Figure 1c and Table S4). Inhibitors were added at various concentrations 4 h after the start of cultivation. The results revealed that S288C displayed greater tolerance to inhibitor treatment. Thus, 50% inhibition of the specific growth rate could not be achieved, even at the highest possible inhibitor concentrations. The treatment of 5 μM of FCCP reduced growth by only 9.7% (Figure 1c). Similarly, growth inhibitions of 19% and 22% were observed for antimycin A and oligomycin, respectively, even at the highest tested concentration of 40 μM (Figure 1c). Although the inhibitor concentrations differed between the MCF7 and S288C cells, these inhibitor concentrations were used consistently throughout the study, as partial growth inhibition was achieved in both cells.

To investigate metabolic rewiring induced by inhibitor treatment, MCF7 cells were cultured under identical conditions. The concentrations of glucose and lactate in the medium were measured over time to calculate the mass balance during log phase (Figure 1d and Table S5). The specific rates for glucose uptake and lactate production in the control culture were determined to be 1.23 ± 0.03 and 2.05 ± 0.02 μmol (10^6^ cells)^−1^ h^−1^, respectively. These rates corresponded to the carbon uptake and production rates of 7.40 ± 0.12 and 6.15 ± 0.06 μCmol (10^6^ cells)^−1^ h^−1^, as glucose and lactate contain six and three carbon atoms per molecule, respectively. When the carbon uptake rate was set at 1.00, the relative number of carbon atoms excreted as lactate was 0.83. The active production of lactate from glucose, even under aerobic conditions, is referred to as aerobic glycolysis, also known as the Warburg effect. FCCP treatment significantly enhanced both carbon uptake and lactate excretion, with relative levels of 1.47 and 1.26, respectively, compared with the control (Figure 1d). These findings indicate that FCCP treatment induces metabolic rewiring characterized by enhanced aerobic glycolysis. Similar increases in aerobic glycolysis were observed after treatment with antimycin A or oligomycin (Figure 1d).

The difference in carbon balance (Diff) between glucose uptake and lactate excretion was further examined. For the control MCF7 cells, the Diff value was calculated to be 0.17 (Figure 1d). Most carbon atoms are consumed for oxidative phosphorylation via the TCA cycle in the mitochondria and for the synthesis of cellular components, such as amino acids and lipids. Analysis of the inhibitor-treated cells showed that the Diff value increased to 0.21 with FCCP treatment and decreased to 0.08 with antimycin A or oligomycin treatment (Figure 1d). The reduction in the Diff value for antimycin A- and oligomycin-treated cells reflects decreased carbon consumption for both the TCA cycle and the biosynthesis of cell components due to inhibition of the respiratory chain. In contrast, the elevated Diff levels in FCCP-treated cells suggested increased flux through the TCA cycle and the respiratory chain. Since FCCP is an uncoupler of the proton gradient in the mitochondria, this activation of the respiratory chain likely serves to maintain MMP.

Similar analysis was performed for S288C cells (Figure 1e and Table S4). The carbon uptake rate for glucose in the control was calculated to be 88 mCmol g DCW^−1^ h^−1^. The carbon consumption attributed to ethanol, CO_2_, and glycerol production accounted for 0.71 (Figure 1e). Active aerobic glycolysis in the S288C cells is also known as the Crabtree effect. Moreover, metabolic rewiring in S288C following inhibitor treatment was nearly identical to that observed in MCF7 cells (Figure 1e).

### 3.2. Control Mechanisms Governing Metabolic Flux Are Shared Between Human Breast Cancer Cells, MCF7, and S. Cerevisiae, S288C

**Figure 2:**
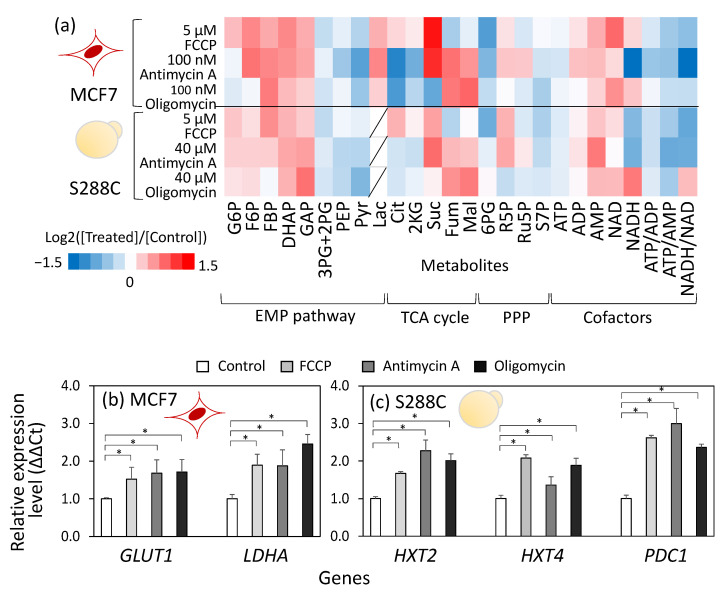
Similar metabolic responses upon respiratory chain inhibitor treatment between human breast cancer cells, MCF7, and *S. cerevisiae*, S288C. (**a**) Metabolic profiles in inhibitor-treated human breast cancer cells, MCF7, and *S. cerevisiae,* S288C. Intracellular metabolites in MCF7 and S288C cells were collected at 3 h and 2 h after inhibitor treatment, respectively, and served for the targeted metabolome analysis. All results were obtained from duplicate or triplicate cultures. The relative amounts of metabolites to the control are shown in heatmap. (**b**,**c**) Effect of FCCP, antimycin A, and oligomycin treatment on gene expression of (**b**) MCF7 and (**c**) S288C cells. RNA was collected from MCF7 and S288C cells at 3 h and 2 h after inhibitor treatment, respectively, and served for the quantitative PCR analysis. The relative expression levels to the control were shown. All results were obtained from triplicate cultures and described as mean ± SD. Asterisks indicate *p*-value < 0.05 by two-sided *t*-test. Red (MCF7) and yellow (S288C) illustrations indicate the cell types used in each experiment.

Metabolic profiling analysis was conducted to estimate the metabolic control mechanisms underlying metabolic rewiring. MCF7 and S288C cells were cultivated under the aforementioned conditions. Cells were collected 3 h and 2 h after inhibitor treatment, respectively, and preserved for targeted metabolome analysis using LC-MS/MS and GC-MS. A metabolic profile dataset including 23 metabolites involved in central carbon metabolism was used for the analysis (Table S6). Figure 2a shows the amount of metabolites relative to control samples.

The metabolic profiles revealed that treatment with FCCP, antimycin A, or oligomycin commonly caused a reduction in the ATP content as well as in the ATP/ADP and ATP/AMP ratios in both MCF7 and S288C cells (Figure 2a). This metabolic state indicated that the inhibition of the respiratory chain decreased the ATP supply. Moreover, the inhibitor treatments commonly induced an increase in metabolites in the upper part of the Embden-Meyerhof-Parnas (EMP) pathway, including fructose-6-phosphate (F6P), fructose-1,6-bisphosphate (FBP), dihydroxyacetone phosphate (DHAP), and glyceraldehyde-3-phosphate (GAP), while inducing a decrease in metabolites in the lower part of the EMP pathway, including 3-phosphoglycerate (3PG) + 2-phosphoglycerate (2PG), phosphoenolpyruvate (PEP), and pyruvate (Pyr). The metabolic profile is a signature of the elevated flux level of the EMP pathway because similar metabolic responses were observed during glucose pulse experiments in *S. cerevisiae* [26]. In these experiments, under glucose-limited conditions, the addition of a high concentration of glucose suddenly elevated metabolic flux in the EMP pathway, leading to the accumulation of upper EMP pathway metabolites, including FBP, an allosteric activator of pyruvate kinase that catalyzes the final step of the EMP pathway [26]. The accumulation of FBP allosterically activates pyruvate kinase, promoting the elimination of intermediates in the lower part of the EMP pathway, including 2PG, 3PG, and PEP [27,28].

In contrast to the common response in the EMP pathway, the metabolite profiles of the TCA cycle differed between inhibitors. In MCF7 cells, FCCP treatment increased the citrate (Cit) and 2-ketoglutarate (2KG) levels, whereas the levels of fumarate (Fum) and malate (Mal) decreased (Figure 2a). Conversely, in cells treated with antimycin A or oligomycin, decreases in Cit and 2KG, and increases in Fum and Mal were observed (Figure 2a). A similar trend was observed in S288C (Figure 2a). These metabolic responses suggest that the entry point of the TCA cycle was upregulated by FCCP treatment and downregulated by antimycin A and oligomycin treatments, which is consistent with the Diff levels in metabolic rewiring (Figure 1d,e).

The mechanisms controlling metabolic rewiring were investigated using gene expression analyses. Quantitative PCR analysis of MCF7 cells conducted 3 h after inhibitor treatment revealed that the expression of *GLUT1*, which is reported to be a highly expressed glucose transporter gene in cancer cell lines [29], increased significantly by 1.5- to 1.7-fold following inhibitor treatment (Figure 2b). Moreover, the expression of *LDHA*, a major isozyme of lactate dehydrogenase (LDH), increased 1.8- to 2.4-fold after inhibitor treatment (Figure 2b). Gene expression analysis of S288C also showed that inhibitor treatment upregulated the expression levels of the high-affinity glucose transporters *HXT2* and *HXT4* [30] and *PDC1*, which encodes the major isoenzyme of pyruvate decarboxylase (PDC), involved in the biosynthesis of ethanol from pyruvate (Figure 2c).

These results are consistent with the metabolome data. The low ATP/AMP ratio in the inhibitor-treated cells likely activates AMP-activated protein kinase (AMPK), a positive regulator of *GLUT1* and *LDHA* expression [31]. Moreover, these findings suggest that the control mechanisms governing metabolic flux in the EMP pathway and the TCA cycle are shared between human breast cancer cells, MCF7, and *S. cerevisiae*, S288C, as evidenced by similar changes in metabolic flux, metabolite accumulation, and gene expression levels.

### 3.3. Constant Mitochondrial Membrane Potential in Live Cells upon Respiratory Chain Inhibitor Treatment

**Figure 3:**
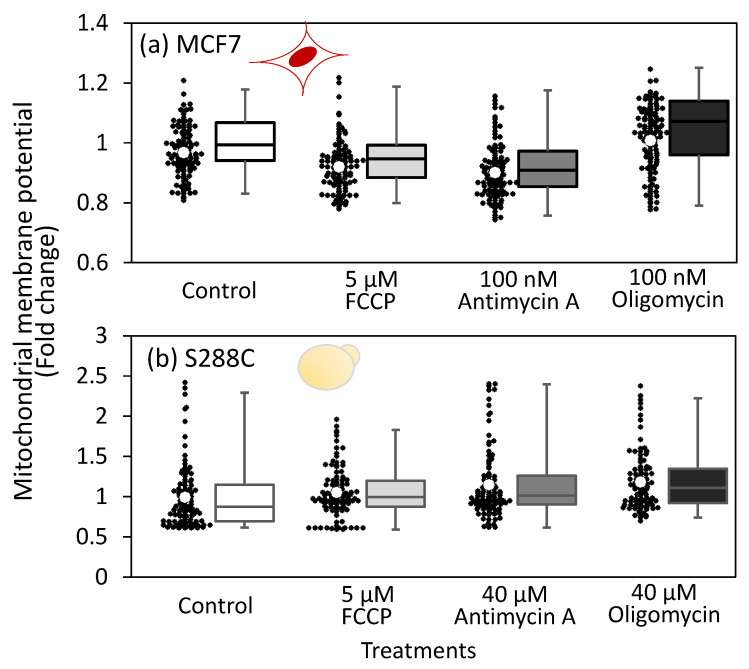
Effect of FCCP, antimycin A, and oligomycin treatment on mitochondrial membrane potential (MMP) levels in live cells. MitoTracker reagent was applied to MCF7 and S288C cells 3 h and 2 h after inhibitor treatment, respectively, followed by fluorescent microscopy analysis. (**a**,**b**) MMP levels in inhibitor treated (**a**) MCF7 and (**b**) S288C cells. Black dots indicate the mean fluorescence intensity of each individual cell, calculated from the fluorescence images. White circles represent the mean intensity levels of all investigated cells relative to the control. A box plot is also used to illustrate the distribution intensity values. Red (MCF7) and yellow (S288C) illustrations indicate the cell types used in each experiment. No significant change in MMP was observed, as determined by a two-sided *t*-test with α = 0.05.

Decreased MMP levels are closely related to cell survival via the induction of apoptosis [32]. Since inhibition of the respiratory chain is expected to affect MMP, we investigated the effect of a respiratory chain inhibitor treatment on MMP. To analyze MCF7 and S288C cells, cultures were incubated for 3 h and 2 h, respectively, before treatment with the MitoTracker reagent for fluorescence imaging analysis (Figure S2). Fluorescence imaging analysis showed no significant changes in the mean fluorescence levels of live cells after inhibitor treatment in either MCF7 or S288C (Figure S3 and Figure 3a,b). While the lack of an effect on MMP by respiratory chain inhibitor treatment was counterintuitive, it can be explained by the low doses of inhibitors and the existence of some mechanisms to maintain MMP. These results suggest that the potential purpose of metabolic rewiring is to maintain the MMP at a constant level despite inhibitor treatment.

### 3.4. Activation of Mitochondrial ATP Consumption by FCCP Treatment

To investigate the mechanisms underlying robust MMP, ATP levels in the cytoplasm and mitochondria of *S. cerevisiae* were measured using the ATP biosensor QUEEN-2m [22]. Two *S. cerevisiae* strains expressing QUEEN-2m in the cytoplasm (YAS002) and mitochondria (YAS003) were generated from the BY18615 strain (Table S1 and Figure S4). For QUEEN-2m expression in the mitochondria, mito-QUEEN-2m with a mitochondrial transport signal was used because its mitochondrial localization was confirmed in the original paper [24].

Both the strains were cultured under identical conditions. The cells were collected during the log phase and adhered to glass plates. Following the inhibitor treatment for 12 min, the fluorescence intensity of each cell line was measured every 3 min for 120 min under a fluorescence microscope. ATP in the cytoplasm was denoted as ATP_Cyto_, and ATP in the mitochondria was denoted as ATP_Mito_.

**Figure 4:**
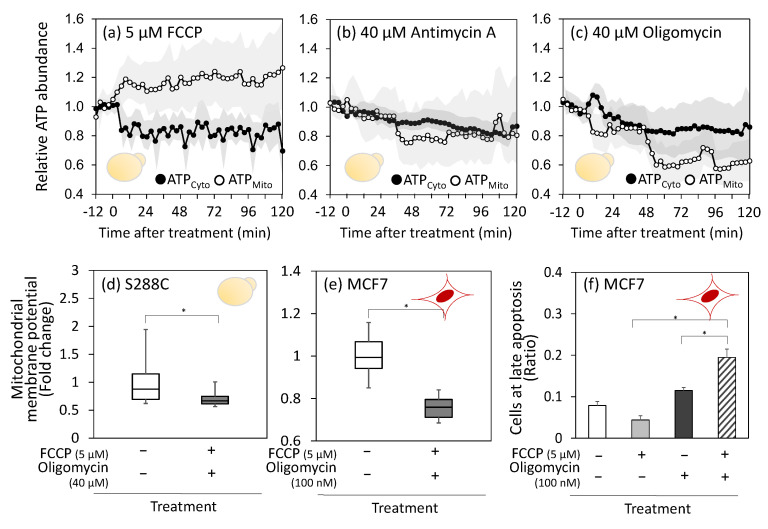
Mitochondrial ATP consumption by FCCP treatment via activation of reverse-mode H^+^/ATPase. (**a**–**c**) ATP abundances in the cytoplasm (ATP_Cyto_) and mitochondria (ATP_Mito_) of *S. cerevisiae* after (**a**) 5 µM FCCP, (**b**) 40 µM antimycin A, and (**c**) 40 µM oligomycin treatment. Cells from two *S. cerevisiae* strains expressing the ATP biosensor QUEEN-2m in the cytoplasm or mitochondria were collected during the log phase and adhered to glass plates. Following the inhibitor treatment for 12 min, the fluorescence intensity of each cell was measured every 3 min using fluorescence microscopy. For each cell, fluorescence intensity at the start of the experiment was set to 1.0. Mean values of more than 30 cells were shown as open and close circles. Gray shadow represents their standard deviations. (**d**,**e**) MMP after co-treatment with FCCP and oligomycin. (**d**) *S. cerevisiae* S288C and (**e**) human breast cancer cells (MCF7) were treated with inhibitors for 2 h and 3 h, respectively, and analyzed using MitoTracker reagent. The box plot represents the distributions of more than 30 cells. (**f**) Induction of apoptosis by the inhibitor treatment. Human breast cancer cells MCF7 were treated with inhibitors for 24 h, and proportion of cells in late apoptosis were determined. Data was presented from triplicate cultures. Asterisks indicate *p*-value < 0.05 by two-sided *t*-test. Red (MCF7) and yellow (S288C) illustrations indicate the cell types used in each experiment.

Treatment with respiratory inhibitors is expected to decrease the mitochondrial ATP production. However, immediately after FCCP treatment, ATP_Mito_ levels increased by approximately 1.2 times, whereas ATP_Cyto_ levels in the cytoplasm decreased by approximately 0.8 of their initial value (Figure 4a). This result suggests downregulation of ATP transport from the mitochondria to the cytosol or activation of reverse ATP transport from the cytosol to the mitochondria. The latter response is plausible because FCCP is known to cause magnesium leakage from mitochondria [33] and mitochondrial calcium accumulation [34], which triggers the activation of the ATP-Mg/Pi transporter and the accumulation of ATP in mitochondria. The accumulated ATP is thought to drive H^+^/ATPase in reverse mode, generating a proton gradient as a proton pump [35]. The Pi generated by the ATPase is expelled back into the cytoplasm by the ATP-Mg/Pi transporter.

In the case of antimycin A treatment, no significant changes were observed in ATP_Cyto_ or ATP_Mito_ levels (Figure 4b). Subsequently, both ATP_Cyto_ and ATP_Mito_ levels gradually decreased in parallel, reaching 0.8 to 0.9 of their initial levels after 120 min. During oligomycin treatment, ATP_Mito_ levels briefly decreased and then dropped again at approximately 50 min to approximately half of the control levels (Figure 4c). The ATP_Cyto_ levels also gradually decreased. A possible interpretation of these behaviors is that mitochondrial ATP levels are maintained by reducing ATP transport from the mitochondria to the cytosol or by reversing ATP transport from the cytosol to the mitochondria.

To investigate the direction of ATP transport, the cells were co-treated with FCCP and oligomycin. Unlike the individual treatments shown in Figure 3, co-treatment with FCCP and oligomycin reduced MMP in S288C cells (Figure 4d and Figure S5). These results suggest that oligomycin inhibits ATP hydrolysis by H^+^/ATPase when treated simultaneously with FCCP and that H^+^/ATPase activity contributes to the maintenance of membrane potential. This decrease in MMP after co-treatment was also observed in MCF7 cells (Figure 4e and Figure S5). These results suggest that ATP transport from the cytoplasm to the mitochondria, coupled with H^+^/ATPase functioning in the reverse mode, contributes to robust MMP upon inhibitor treatment.

Next, we investigated the relationship between the decreased MMP levels and programmed cell death. MCF7 cells were collected 24 h after inhibitor treatment and subjected to an apoptosis assay. The results showed that compared to individual FCCP or oligomycin treatments, co-treatment with FCCP and oligomycin increased the proportion of cells in the late stages of apoptosis by approximately 1.5–3.0-fold (Figure 4f). This suggests that the decrease in MMP caused by the co-treatment triggers cellular apoptosis. However, co-treatment failed to induce cell death in S288C cells, probably because of the lower susceptibility of S288C cells to inhibitors (Figure 1c).

### 3.5. Mitochondrial Membrane Potential Under Hypoxic or Low Oxygen Conditions

**Figure 5:**
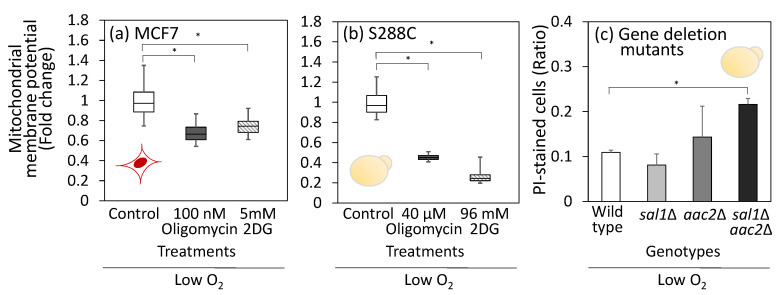
Role of reverse mode H^+^/ATPase under low oxygen condition. (**a**,**b**) Effect of oligomycin and 2-deoxyglucose (2DG) treatment on MMP levels**.** (**a**) MCF7 cells were cultured in the medium containing the inhibitors for 24 h at 1% O_2_, 5% CO_2_, and 37 °C using a low-oxygen incubator. (**b**) For S288C, cells were cultured in the 1 mL of SD medium containing inhibitors and placed in an anaerobic chamber at 30 °C for 24 h. After treatment with MitoTracker reagent, fluorescence was observed while maintaining hypoxic conditions on a glass plate in the low-oxygen incubator. Box plots represent distribution of MMP for more than 30 cells. Data were obtained from triplicated cultures. (**c**) Effect of mitochondrial ATP carrier gene deletions on cell viability under the anaerobic condition. Wild-type, *sal1*Δ, *aac2*Δ, and *sal1*Δ*aac2*Δ strains were cultured under the aforementioned anaerobic conditions for 24 h. Cell were stained with propidium iodide (PI) to examine dead cell rates through microscopic observation. Data were obtained from duplicated cultures. Asterisks indicate *p*-value < 0.05 by two-sided *t*-test. Red (MCF7) and yellow (S288C) illustrations indicate the cell types used in each experiment.

Under hypoxic conditions, the activity of the electron transport chain is reduced because of the lack of oxygen as an electron acceptor, suggesting that H^+^/ATPase operates in the reverse mode to maintain MMP [35,36,37]. To investigate the role of H^+^/ATPases, MCF7 and S288C cells were treated with oligomycin under hypoxic conditions. Unlike the normoxic conditions shown in Figure 3, the results for MCF7 cells showed that the MMP decreased by 27% with 100 nM oligomycin treatment in a hypoxic environment (Figure 5a and Figure S6). These results suggested that ATP hydrolysis by H^+^/ATPase was inhibited by oligomycin under hypoxic conditions. A similar decrease in MMP was observed in oligomycin-treated S288C (Figure 5b and Figure S6).

Moreover, to assess its dependence on ATP transport from the cytoplasm to the mitochondria, 2-deoxyglucose (2DG) was used to inhibit the EMP pathway. The MMP of both MCF7 and S288C cells decreased significantly after 2DG treatment (Figure 5a,b). These results support the hypothesis that the reverse mode of H^+^/ATPase and ATP derived from substrate-level phosphorylation contribute to the maintenance of MMP under low-oxygen conditions.

ADP/ATP carrier proteins in the inner mitochondrial membrane play crucial roles in ATP transport between the cytoplasm and mitochondria. Humans and *S. cerevisiae* commonly have three isoforms: *AAC1*, *AAC2*, and *AAC3* genes for the case of *S. cerevisiae.* It has been reported that Aac2 makes the largest contribution to ATP transport from the cytosol to the mitochondria [38]. Additionally, the calcium-dependent ATP-Mg/Pi carrier Sal1 contributes to the cytosol-to-mitochondrial ATP transport [39]. In this study, single and double gene deletion strains including *sal1*Δ, *aac2*Δ, and *sal1*Δ*aac2*Δ were constructed and cultured under the anaerobic conditions for 24 h. Cells were stained by propidium iodide to assess the dead cell rate (Figure 5c). In the wild-type strain, the dead cell rate was 10.9%; the *sal1*Δ and *aac2*Δ strains showed no significant increase in dead cell rate, whereas the *sal1*Δ*aac2*Δ double knockout strain exhibited significant increase, with a dead cell rate of 19.6% (Figure 4c). These results suggest that ATP transport from the cytoplasm to the mitochondria plays an important role in maintaining the cell viability of *S. cerevisiae* under anaerobic conditions.

## 4. Discussion

This study proposes that ATP supply from the cytosol to the mitochondria is a common role of aerobic glycolysis in MCF7 and S288C cells to prevent programmed cell death by maintaining MMP. A common role was suggested by the finding that metabolic responses to oxidative phosphorylation dysfunction caused by inhibitor treatment were similar between MCF7 and S288C cells. Both cells showed metabolic rewiring toward substrate-level phosphorylation (Figure 1d,e). Similar patterns were observed in metabolic profile changes, including the accumulation of FBP and lower ATP/ADP ratios (Figure 2a). Expression of genes encoding glucose transporters and enzymes in the final steps of substrate-level phosphorylation was upregulated (Figure 2b,c). The similar metabolic response can be partly explained by a conserved signaling mechanism in *S. cerevisiae* and human cancer cells. A low ATP/AMP ratio upregulates the EMP pathway via AMPK [31]. FBP binds to Cdc25/Sos1 and activates the EMP pathway via the cAMP/PKA pathways [40].

The similar metabolic response suggests that mitochondrial oxidative phosphorylation and cytoplasmic substrate-level phosphorylation are interconnected via a common mechanism in both MCF7 and S288C cells. The data presented in this study suggest that the ATP supply from the cytosol to the mitochondria is an additional role of aerobic glycolysis. As its name implies, mitochondrial H^+^/ATPase can hydrolyze ATP and acts as a proton pump to generate membrane potential [35]. The reverse mode of H^+^/ATPase activity was inferred from the observation that MMP remained unchanged in inhibitor-treated MCF7 and S288C cells (Figure 3). In *S. cerevisiae*, the cytoplasmic ATP levels decreased, whereas the mitochondrial ATP levels increased after FCCP treatment (Figure 4a). Furthermore, simultaneous treatment with FCCP and oligomycin decreased MMP in both *S. cerevisiae* S288C and human breast cancer cells MCF7 (Figure 4d,e). Moreover, under hypoxic conditions, the MMP levels decreased following oligomycin treatment or EMP pathway inhibition in both cell types (Figure 5a,b).

Similar observations have been made in other mammalian normal and cancer cells. For example, the capacity of the reverse mode of ATP synthase to generate MMP is increased in the brown fat tissue of cold-adapted mice [41]. Furthermore, the complete inhibition of complex I by rotenone still maintains MMP in nerve cells [42,43]. MMP has also been preserved in various human cells lacking mitochondrial DNA [44]. The treatment of mitochondria, derived from patients with mitochondrial dysfunction diseases, with oligomycin results in the loss of membrane potential [45]. In IF1-deficient 143C cells, co-treatment with FCCP and oligomycin decreased membrane potential [46]. Furthermore, the inhibition of the reverse mode of H^+^/ATPase in cancer cells, such as 143B cells, reduces MMP [38]. In cardiac cells, under ischemic or hypoxic conditions, mitochondria consume ATP through H^+^/ATPase, operating in the reverse mode [36,37]. Mitochondrial ATP consumption has been implicated in cancer and other diseases [47,48].

The results of this study also indicate that MMP maintenance is a critical aspect of cell survival. Co-treatment of MCF7 cells with FCCP and oligomycin resulted in low MMP (Figure 4e) and the induction of apoptosis (Figure 4f). Moreover, the *sal1Δaac2Δ* double knockout strain of *S. cerevisiae* repressing ATP transport from the cytosol to the mitochondria exhibited increased dead cell rate under anaerobic conditions (Figure 5c). A growing body of evidence supports the notion that MMP is closely associated with programmed cell death [49]. For example, treatment with the complex I inhibitor rotenone has been reported to induce apoptosis in MCF7 [50], and the loss of membrane potential can trigger apoptosis [51]. An unstable membrane potential amplifies apoptosis triggered by other factors [32].

Based on these results, the ATP supply to mitochondria appears to play a key role in aerobic glycolysis, and this role is conserved in both cancer and yeast cells. This function seems to be not always active and becomes significant under hypoxic or unstable oxygen supply conditions, where the membrane potential cannot be stably maintained by the activity of the electron transport chain [36,37,39].

For example, *S. cerevisiae* specializes in niches with high sugar concentrations, such as flower nectar [52]. High-sugar and low-oxygen supply environments facilitate rapid proliferation via active metabolism, which quickly depletes dissolved oxygen and creates an anaerobic microenvironment [53]. Moreover, for floating cells, the activity of the electron transport chain may become unstable owing to fluctuating concentrations of dissolved oxygen. Under such conditions, constant activation of substrate-level phosphorylation in the cytosol provides a strategic advantage by acting as an emergency mechanism to meet sudden ATP demands from the mitochondria.

Cancer cells are survivors that resist diverse pressure from the immune system to induce cell death [54]. Cancer cells can also proliferate under hypoxic conditions with an unstable oxygen supply [47]. To endure such adverse conditions, constant activation of substrate-level phosphorylation is essential for maintaining MMP at a safe level, thereby preventing the onset of apoptosis.

More direct evidence is required to confirm the additional role of aerobic glycolysis. The measurement of apoptosis-related metabolites and enzymes, such as reactive oxygen species and superoxide dismutase, will support the relationship between the reverse mode of H^+^/ATPase, MMP, and programmed cell death. ATP transport from the cytosol to the mitochondria in the reverse mode of H^+^/ATPase can be verified by tracer experiments using stable isotope labels. However, the rapid turnover of ATP and cellular component-specific analyses are the current technical bottlenecks.

Although its origin remains unclear [55], aerobic glycolysis and its role in supplying ATP to mitochondria may be an evolutionarily ancient mechanism, because apoptosis is a universal mechanism in eukaryotes [56,57]. Aerobic glycolysis and reverse-mode ATPases have been identified in various species and cell types. For example, various mammalian stem cells [58,59] and Drosophila macrophages rely on aerobic glycolysis [60]. When a protist infects primary human macrophages, mitochondrial H^+^/ATPase switches to ATP hydrolase activity and prevents macrophage cell death [61]. As previously mentioned, aerobic glycolysis plays several roles in various biological processes. A more detailed investigation of the additional roles of aerobic glycolysis will lead to a better understanding of the biological significance and clinical and industrial applications of the Warburg effect in mammalian cancer cells and the Crabtree effect in *S. cerevisiae*.

## Data Availability

The data presented in this study are available upon request from the corresponding author.

## Supplementary Materials

The following supporting information can be downloaded at: https://www.mdpi.com/article/10.3390/metabo15070461/s1, Figure S1: Vector map of pAS2; Figure S2: Fluorescent imaging of mitochondrial membrane potential using MitoTracker reagent; Figure S3: Fluorescent imaging of mitochondrial membrane potential of inhibitor-treated human breast cancer cell, MCF7, and *S. cerevisiae*, S288C; Figure S4: Localized expression of QUEEN-2m in cytosol and mitochondria; Figure S5: Fluorescent imaging of mitochondrial membrane potential of human breast cancer cell, MCF7, and *S. cerevisiae*, S288C, treated with FCCP and oligomycin; Figure S6: Fluorescent imaging of mitochondrial membrane potential of human breast cancer cell, MCF7, and *S. cerevisiae*, S288C, treated with oligomycin under hypoxic conditions; Table S1: *S. cerevisiae* strains and plasmids used in this study; Table S2: Primers for qPCR; Table S3: Primers to construct pAS1 and pAS2; Table S4: Specific rates for cell proliferation, glucose uptake, and ethanol and glycerol production of inhibitor-treated *S. cerevisiae* (S288C) cells during log-phase; Table S5: Specific rates for cell proliferation, glucose uptake, and lactate production of inhibitor-treated human breast cancer (MCF7) cells during log-phase; Table S6: Metabolic profile data obtained from inhibitor-treated S288C and MCF7 cells.
