# Supplementary material for: ATP Supply from Cytosol to Mitochondria Is an Additional Role of Aerobic Glycolysis to Prevent Programmed Cell Death by Maintenance of Mitochondrial Membrane Potential
Source: Metabolites. 2025 Jul 7;15(7):461. doi: 10.3390/metabo15070461 (PMC12299498; doi:10.3390/metabo15070461)
Supplement: Supplementary file 1 [file metabolites-15-00461-s001.zip › metabolites-3688469-supplementary.pdf]

Supplementary figures and tables

ATP supply from cytosol to mitochondria is an additional role of aerobic glycolysis to prevent programmed cell death by maintenance of mitochondrial membrane potential

Akane Sawai, Takeo Taniguchi, Kohsuke Noguchi, Taisuke Seike, Nobuyuki Okahashi, Masak Takaine Fumio Matsuda*

Figure S1 Vector map of pAS2

Figure S2 Fluorescent imaging of mitochondrial membrane potential using MitoTracker reagent.

Figure S3 Fluorescent imaging of mitochondrial membrane potential of inhibitor treated human breast cancer cell, MCF7 and *S. cerevisiae* S288C.

Figure S4 Localized expression of QUEEN-2m in cytosol and mitochondria.

Figure S5 Fluorescent imaging of mitochondrial membrane potential of human breast cancer cell, MCF7 and *S. cerevisiae* S288C treated with FCCP and oligomycin.

Figure S6 Fluorescent imaging of mitochondrial membrane potential of human breast cancer cell, MCF7 and *S. cerevisiae* S288C treated with oligomycin under hypoxic conditions.

Table S1 *Saccharomyces cerevisiae* strains and plasmids used in this study

Table S2 Primers for qPCR

Table S3 Primers to construct pAS1 and pAS2

Table S4 Specific rates for cell proliferation, glucose uptake, ethanol and glycerol production of inhibitor treated *S. cerevisiae* (S288C) cells during log-phase.

Table S5 Specific rates for cell proliferation, glucose uptake, and lactate production of inhibitor treated human breast cancer (MCF7) cells during log-phase.

Table S6 Metabolic profile data obtained from inhibitor treated S288C and MCF7 cells

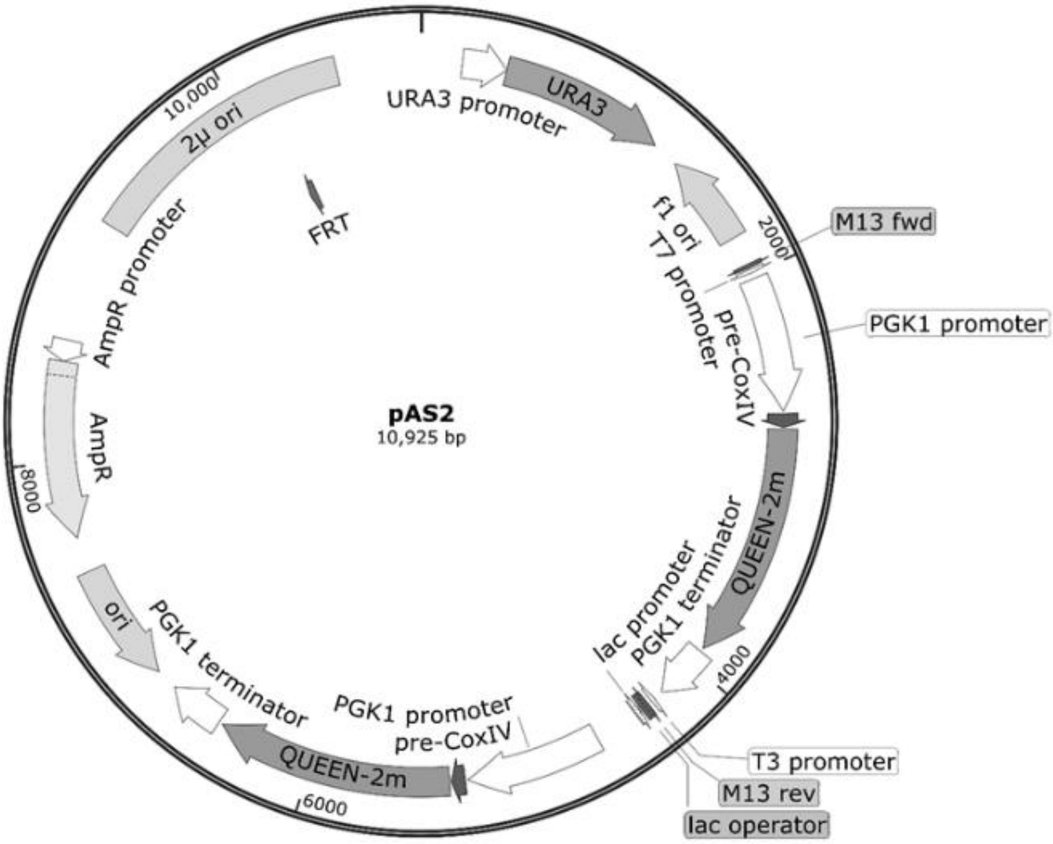

Figure S1 Vector map of pAS2

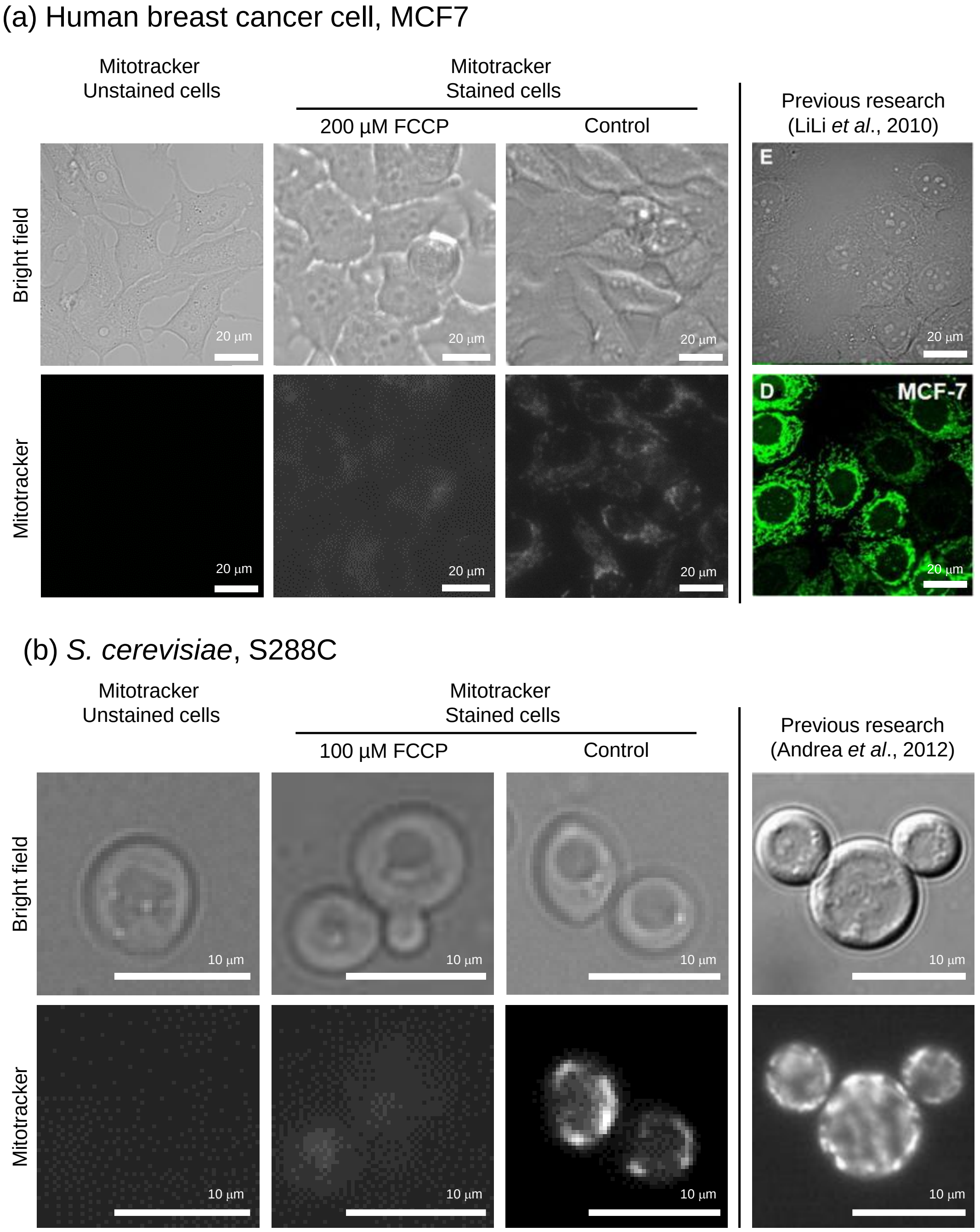

Figure S2 Fluorescent imaging of mitochondrial membrane potential using MitoTracker reagent. MitoTracker reagent was treated to (a) MCF-7 and (b) S288C at 3 and 2 hours after inhibitor treatment, respectively, and served for the fluorescent microscopy.

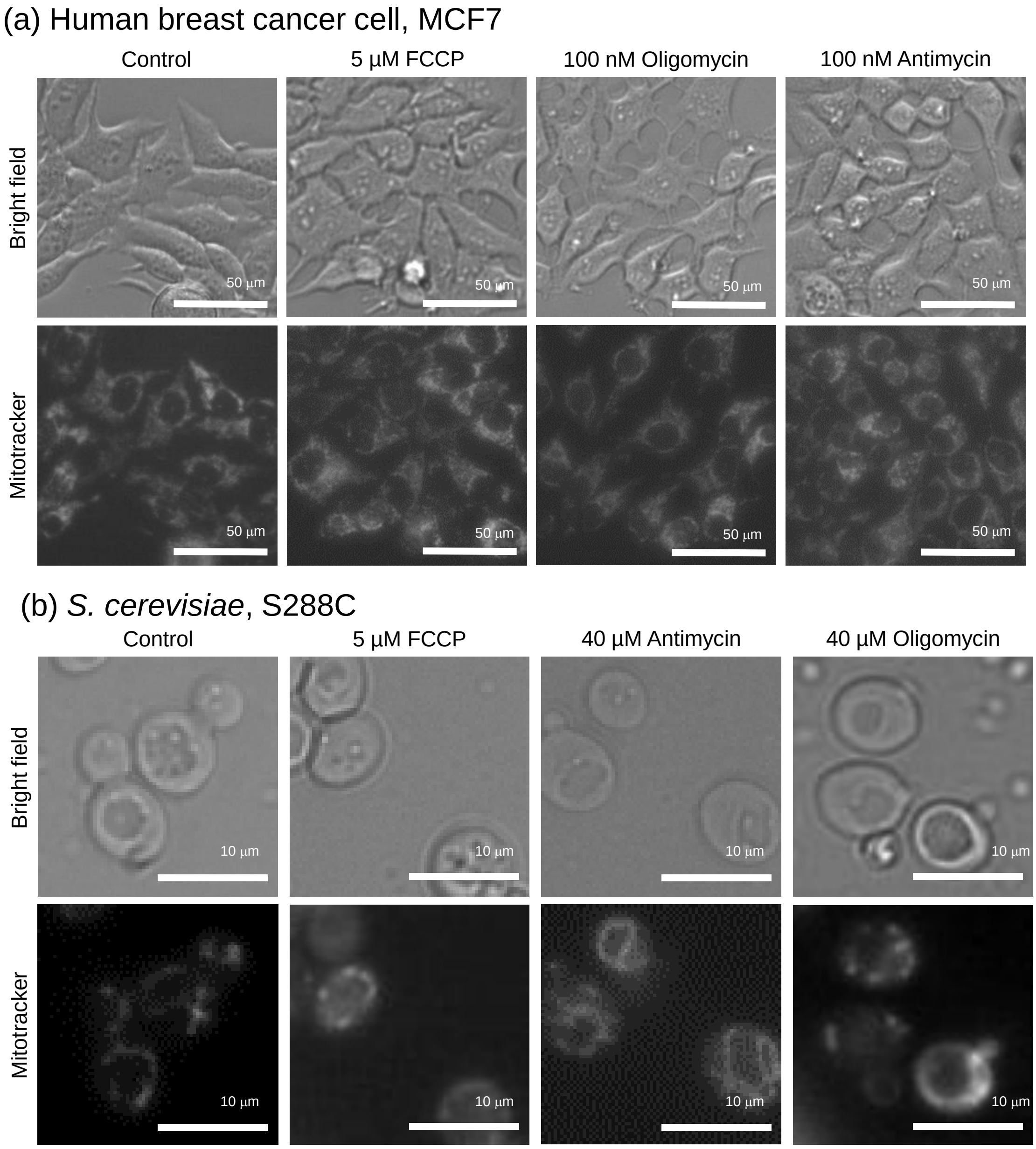

Figure S3 Fluorescent imaging of mitochondrial membrane potential of inhibitor treated human breast cancer cell, MCF7 and *S. cerevisiae* S288C. MitoTracker reagent was treated to (a) MCF-7 and (b) S288C cells at 3 and 2 hours after FCCP, antimycin, and oligomycin treatment, respectively, and served for the fluorescent microscopy.

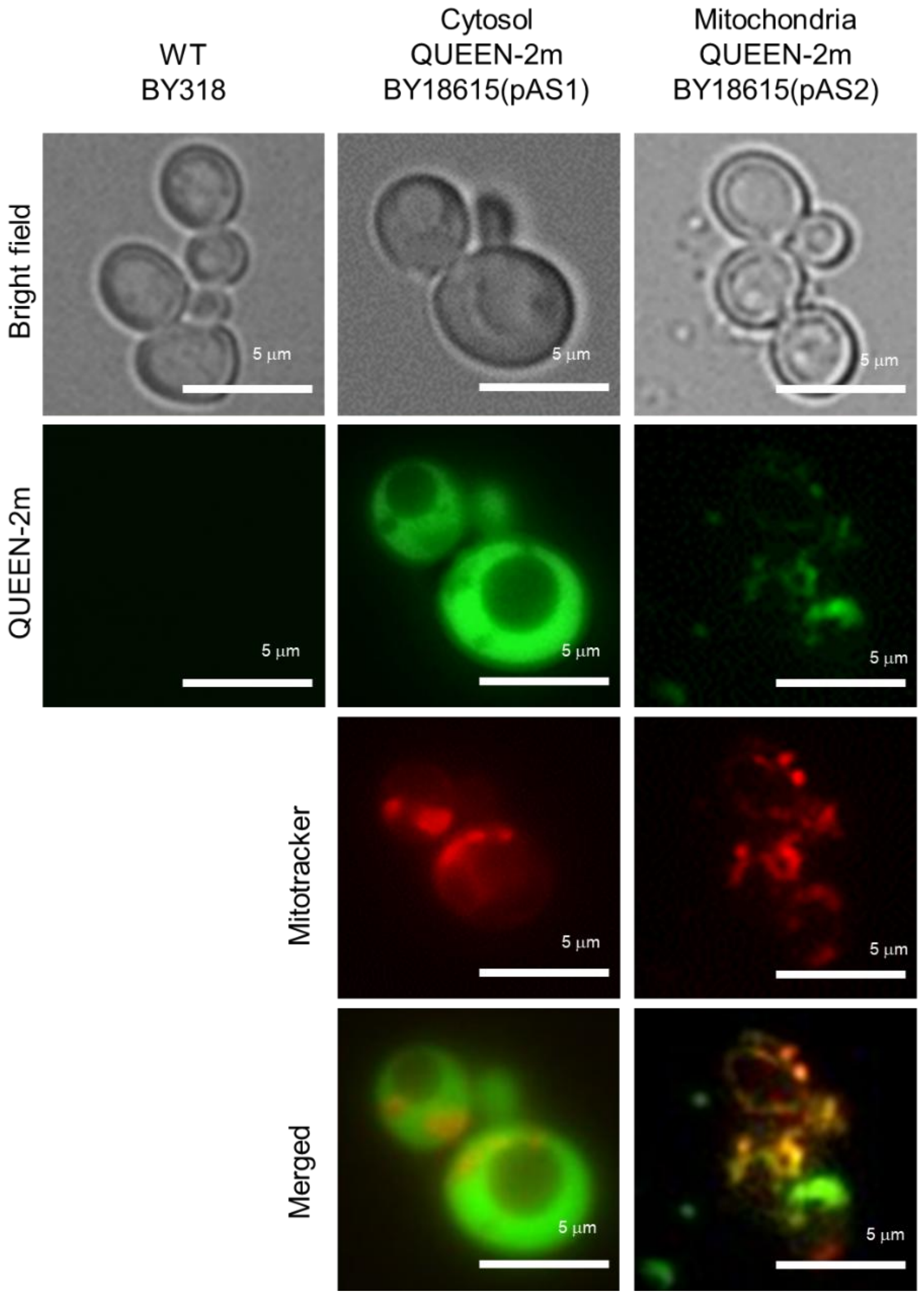

Figure S4 Localized expression of QUEEN-2m in cytosol and mitochondria of *S. cerevisiae* S288C. Images obtained using blight light, a green fluorescence filter, and a red fluorescence filter are shown.

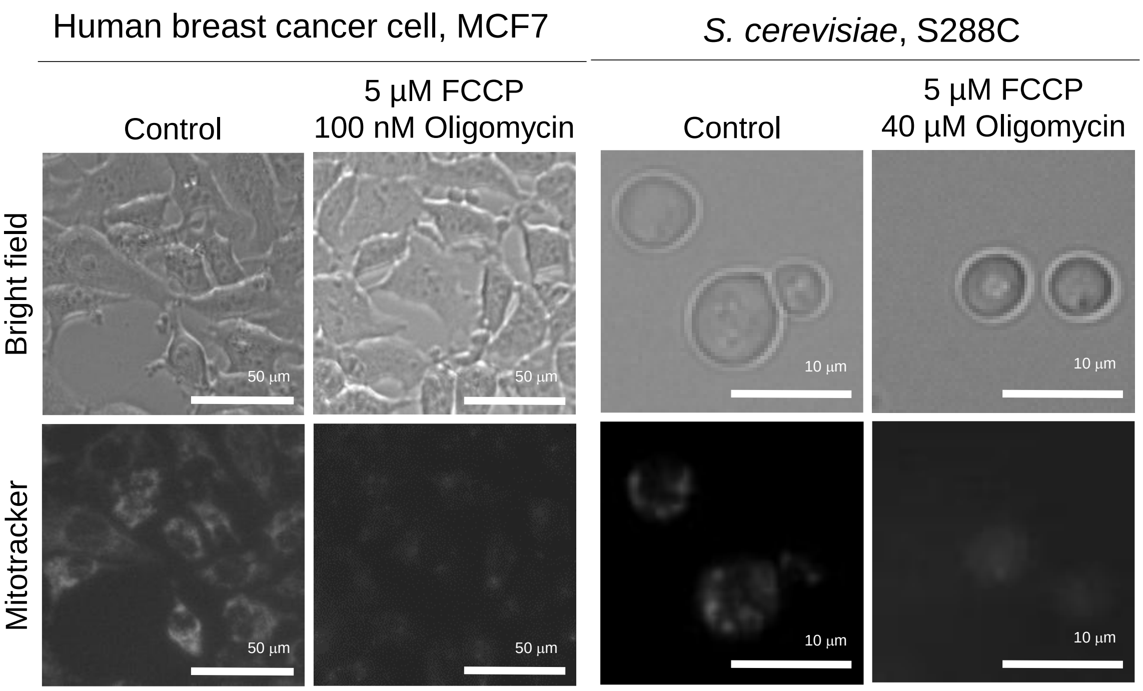

Figure S5 Fluorescent imaging of mitochondrial membrane potential of human breast cancer cell, MCF7 and *S. cerevisiae* S288C treated with FCCP and oligomycin. MitoTracker reagent was treated to (a) MCF-7 and (b) S288C at 3 and 2 hours after the co-treatment of FCCP and oligomycin, respectively, and served for the fluorescent microscopy.

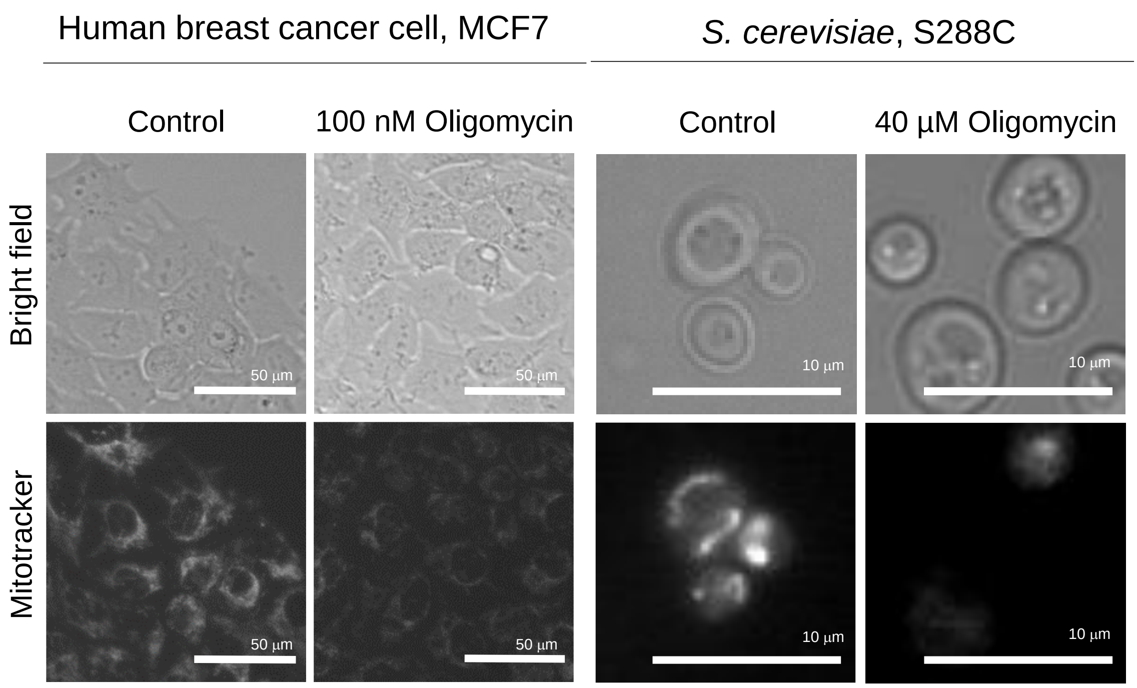

Figure S6 Fluorescent imaging of mitochondrial membrane potential of human breast cancer cell, MCF7 and *S. cerevisiae* S288C treated with oligomycin under hypoxic conditions. MitoTracker reagent was treated to (a) MCF-7 and (b) S288C cultured under low oxygen conditions with oligomycin and served for fluorescent microscopy.

Table S1 *Saccharomyces cerevisiae* strains and plasmids used in this study

Strains	Genotype	Sources
S288C (BY27002)	*MATα mal SUC2*	National BioResource Project (NBRP)
BY18615	*MATα, ura3*	NBRP
YAS001	BY18615[pGK426]	
YAS002	BY18615[pAS1]	
YAS003	BY18615[pAS2]	
**Plasmids**	**Description**	**Ref**
pGK426	Yeast multi-copy type single-gene expression vector containing PGK1 promoter, PGK1 terminator, 2μ origin, and *URA3* marker	[17]
pGK426-QUEEN-2m	pGK426, expression of codon-optimized QUEEN-2m by PGK1 promoter	This study
pAS1	pGK426, an open reading frame for expression of codon-optimized QUEEN-2m with the mitochondrial targeting signal derived from *S. cerevisiae* Cox4 at *N*-terminus by PGK1 promoter	This study
pAS2	pGK426, two open reading frames for expression of codon-optimized QUEEN-2m with the mitochondrial targeting signal derived from *S. cerevisiae* Cox4 at *N*-terminus by PGK1 promoter	This study

Table S2 Primers for qPCR

Genes	Primer sequences (5' to 3') (F: Forward and R: Reverse)
For *S. cerevisiae*, S288C	
ACT1	F：ACATCGTTATGTCCTTGGT
	R：CCACCAATCCAGACGGAGTA
HXT2	F：CTTCGCATCCACTTTCGTG
	R：AATCATGACGTTACCGGCAGCC
HXT4	F：ATGGAGAGTTCCATTAGGTCTAGG
	R：ATAACAGCTGGATCGTCTGCGC
PDC1	F：CTTACGCCGCTGATGGTTA
	R：GGCAATACCGTTCAAAGCAG
CIT2	F：CCTCCAGTTGGCTTATCGTG
	R：CTGAAACGAATACCGTCTTCTG
CAT2	F：CCGTGGTTAAATTCATCGAAGC
	R：ACTAAAGAAGACGGATCTGTGG
FBP26	F：GGAGATGTGCTACCGATATCC
	R：GCCCACACGGCATACTTTTC
For human breast cancer cell, MCF7	
GAPDH	F：ATGGAAATCCCATCACCATCTT
	R：CGCCCCACTTGATTTTGG
GLUT1	F：AGGTGATCGAGGAGTTCTAC
	R：TCAAAGGACTTGCCCAGTTT
PFKP	F：GGGCCAAGGTGTACTTCATC
	R：TGGAGACACTCTCCCAGTCG
LDHA	F：GGACTTGGCAGATGAACTTG
	R：TCAGAGAGACACCAGCAACA
CPT1	F：CCTCCAGTTGGCTTATCGTG
	R：TTCTTCGTCTGGCTGGACAT
CPT2	F：GCAGATGATGGTTGAGTGCTCC
	R：AGATGCCGCAGAGCAAACAAGTG
PFKB4	F：GGGTGCCTCTTGGCCTTAAA
	R：GCCCACACGGCATACTTTTC

Table S3 Primers to construct pAS1 and pAS2

Genes	Primer sequences
Queen-2m_Cox4N_invF	CAGCCACAAGAACTTTGTGTAGCTCTAGATATCTGCTTATGAAGACCGTTAAGGTTAACATCACTAC
Queen-2m_Cox4N_invR	GCTTGAAAAATCTTATAGATTGACGTAGTGAAAGCATGTCGACGCTAGCGTTTTATATTTGTTG
AS1 fusion-F1	GCGGTAATACGGTTATCCACAGAATC
AS1 fusion-R1	CTTTGAGTGAGCTGATACCGCTC
AS1 QUEEN-F1	TCAGCTCACTCAAAGAAAGATGCCGATTTGGGCG
AS1 QUEEN-R1	TAACCGTATTACCGCAACGCAGAATTTTCGAGTTATTAAACTTAAAATACGC

Table S4 Specific rates for cell proliferation, glucose uptake, ethanol and glycerol production of inhibitor treated *S. cerevisiae* (S288C) cells during log-phase.

	Specific rates			
Inhibitors	Cell proliferation (h^{-1})	Glucose uptake (mmol (gDCW h)$^{-1}$)	Ethanol production (mmol (gDCW h)$^{-1}$)	Glycerol production (mmol (gDCW h)$^{-1}$)
Control	0.363±0.011	14.72±0.52	20.71±0.76	0.17±0.01
5 µM FCCP	0.328±0.005*	21.02±0.39*	31.08±0.42*	0.18±0.00
40 µM antimycin	0.312±0.012*	18.07±1.40*	29.13±2.73*	0.34±0.03*
40 µM oligomycin	0.315±0.010*	17.72±1.21*	28.90±1.86*	0.26±0.02*

The specific rates were determined from the growth curve. All results were obtained from triplicate cultures and described as Mean ±SD. Asterisks indicate p-value < 0.05 by two-sided t-test.

DCW: dry cell weight

Table S5 Specific rates for cell proliferation, glucose uptake, and lactate production of inhibitor treated human breast cancer (MCF7) cells during log-phase.

	Specific rates		
Inhibitors	Cell proliferation (h^{-1})	Glucose uptake (nmol (10^6cells h)$^{-1}$)	Lactate production (nmol (10^6cells h)$^{-1}$)
Control	0.0349±0.0017	1237±28	2053±21
5 μM FCCP	0.0194±0.0015*	1822±250*	3114±222*
100 nM antimycin	0.0201±0.0032*	1696±91*	3188±170*
100 nM oligomycin	0.0191±0.0059*	1756±124*	3316±184*

The specific rates were determined from the growth curve. All results were obtained from triplicate cultures and described as Mean ±SD. Asterisks indicate p-value < 0.05 by two-sided t-test.

Table S6 Metabolic profile data obtained from inhibitor treated S288C and MCF7 cells

	S. cerevisiae, S288C			Human breast cancer cell, MCF7		
	Log2(Treated/Control)					
	FCCP	Antimycin A	Oligomycin	FCCP	Antimycin A	Oligomycin
G6P	0.333	0.270	0.156	0.381	-0.070	-0.176
F6P	0.183	0.268	0.198	0.700	0.825	-0.051
FBP	0.660	0.278	-0.037	0.498	0.704	0.772
DHAP	0.437	0.511	0.450	0.696	0.635	0.394
GAP	0.292	0.558	0.832	0.482	0.514	0.273
3PG+2PG	-0.363	-0.175	-0.234	-0.343	-0.117	-0.259
PEP	-0.061	-0.402	-0.382	-0.133	-0.558	-0.077
Pyr	-0.125	-0.420	-0.739	-0.432	-0.972	-0.585
Lactate	nd	nd	nd	0.461	0.703	0.265
Citrate	0.452	-0.257	-0.233	0.230	-1.271	-1.046
2KG	0.077	-0.310	-0.108	0.118	-0.826	-0.400
Succinate	0.545	0.863	0.075	1.396	1.247	-0.900
Fumarate	-0.426	0.401	0.647	-0.500	0.832	0.790
Malate	0.014	0.345	0.773	-0.131	0.622	0.900
6PG	-0.845	-0.134	0.032	-1.016	-0.741	-0.346
R5P	0.452	0.508	-0.337	0.162	0.326	-0.006
Ru5P	-0.197	0.120	-0.192	-0.217	0.316	-0.191
S7P	-0.471	-0.362	-0.216	-0.022	-0.262	-0.501
ATP	-0.182	-0.148	-0.152	-0.060	-0.235	-0.067
ADP	0.069	0.158	0.034	0.156	0.359	-0.012
AMP	0.337	0.783	0.399	0.482	0.390	0.254
NAD	0.195	0.012	0.398	0.880	0.207	0.650
NADH	-0.718	-0.811	0.785	0.222	-2.453	0.327
ATP/ADP	-0.294	-0.293	-0.176	-0.215	-0.583	-0.059
ATP/AMP	-0.525	-0.870	-0.522	-0.545	-0.626	-0.319
NADH/NAD	-0.901	-0.856	0.385	-0.660	-2.664	-0.327

	S. cerevisiae, S288C			Human breast cancer cell, MCF7		
	Two-sided t test (p-value)					
	FCCP	Antimycin A	Oligomycin	FCCP	Antimycin A	Oligomycin
G6P	0.005	0.481	0.422	0.043	0.577	0.203

F6P	0.042	0.521	0.198	0.139	0.053	0.755
FBP	0.366	0.241	0.656	0.045	0.004	0.050
DHAP	0.109	0.168	0.327	0.156	0.010	0.002
GAP	0.408	0.032	0.017	0.094	0.205	0.367
3PG+2PG	0.407	0.672	0.584	0.029	0.222	0.121
PEP	0.924	0.655	0.177	0.336	0.114	0.387
Pyr	0.549	0.085	0.525	0.346	0.041	0.031
Lactate	nd	nd	nd	0.310	0.047	0.375
Citrate	0.002	0.038	0.068	0.146	0.000	0.014
2KG	0.846	0.091	0.672	0.294	0.008	0.021
Succinate	0.093	0.007	0.508	0.004	0.001	0.037
Fumarate	0.307	0.185	0.009	0.016	0.014	0.028
Malate	0.934	0.032	0.010	0.106	0.013	0.017
6PG	0.003	0.647	0.896	0.090	0.002	0.023
R5P	0.187	0.082	0.385	0.565	0.099	0.962
Ru5P	0.270	0.703	0.062	0.337	0.316	0.141
S7P	0.005	0.070	0.068	0.925	0.147	0.116
ATP	0.256	0.332	0.073	0.139	0.186	0.457
ADP	0.767	0.603	0.831	0.043	0.169	0.919
AMP	0.104	0.411	0.124	0.002	0.026	0.032
NAD	0.779	0.863	0.006	0.000	0.096	0.039
NADH	0.476	0.396	0.042	0.117	0.001	0.251
ATP/ADP	0.299	0.158	0.298	0.006	0.128	0.494
ATP/AMP	0.012	0.125	0.049	0.013	0.121	0.013
NADH/NAD	0.000	0.407	0.101	0.031	0.000	0.125
